# Effects of Whole‐Body Electromyostimulation on the Thickness of Appendicular and Respiratory Muscles, Functionality, and Frailty in Patients From a Transitional Care Unit: A Randomized Clinical Trial Protocol

**DOI:** 10.1002/pri.70199

**Published:** 2026-03-16

**Authors:** André Luis Pinheiro Borges, Antônio Anderson Ramos de Oliveira, Juliana Nogueira Coelho, Antônio Riquelme Martins Negreiros, Aparecida Pereira Holanda Almeida Sampaio, Danielle Pessoa Lima, Miguel Ângelo Nobre e Souza, Jarbas de Sá Roriz Filho

**Affiliations:** ^1^ Medical Sciences Graduate Program (PPGCM) Federal University of Ceará (UFC) Fortaleza Ceará Brazil; ^2^ Federal University of Ceará (UFC) Fortaleza Ceará Brazil; ^3^ University of Fortaleza (UNIFOR) Fortaleza Ceará Brazil; ^4^ Walter Cantídio University Hospital Brazilian Company of Hospital Services (Ebserh) Federal University of Ceará (UFC) Fortaleza Brazil; ^5^ Department of Internal Medicine, Division of Geriatrics Federal University of Ceará Fortaleza Ceará Brazil

**Keywords:** electromyostimulation, frailty, muscle ultrasound, physiotherapy

## Abstract

**Background:**

Frailty is highly prevalent among recently hospitalized adults and is associated with functional decline, dependency, and increased mortality. Although conventional physiotherapy is routinely applied in transitional care settings, its effectiveness may be limited in frail individuals with low tolerance to exercise. Whole‐body electromyostimulation (WB‐EMS) has emerged as a potential adjunct intervention to enhance muscle preservation and functional recovery in this population. The aim of this study was to evaluate the effects of WB‐EMS combined with conventional physiotherapy on muscle thickness, functional capacity, and frailty in patients admitted to a transitional care unit.

**Methods:**

This randomized, assessor‐blinded clinical trial will include 62 frail adults (≥ 50 years; Tilburg Frailty Indicator ≥ 5), randomized to receive either WB‐EMS plus physiotherapy or physiotherapy alone for 6 weeks. WB‐EMS will be applied twice weekly (20 min/session), concurrently with standardized low‐load exercises. The primary outcome is the change in rectus femoris muscle thickness assessed by B‐mode ultrasonography. Secondary outcomes include diaphragm and forearm muscle thickness, functional capacity (Barthel Index), and frailty (Tilburg Frailty Indicator). Outcomes will be assessed at baseline and post‐intervention.

**Conclusion:**

This trial will provide evidence on the feasibility and physiological effects of WB‐EMS as an adjunct to physiotherapy in frail patients undergoing transitional care, with potential implications for rehabilitation strategies in post‐hospitalization settings.

**Trial Registration:**

REBEC, RBR‐6DQ3QGN. Registered on 01 November 2024

## 1 Introduction

Hospitalization is a critical event in the trajectory of frailty, often accelerating muscle loss, physical inactivity, and functional decline. These changes substantially increase dependency, prolong recovery, and raise the risk of adverse outcomes after discharge (Cunha et al. 2019). Transitional Care Units (TCUs) have been designed to bridge acute hospital care and community reintegration, offering short‐term rehabilitation and clinical monitoring during a vulnerable recovery phase (Krassikova et al. 2025).

Exercise‐based interventions are recognized as effective strategies for mitigating frailty (Wan et al. 2025; Sirikul et al. 2024). However, frail and recently hospitalized individuals frequently exhibit limited tolerance to conventional exercise programs, reducing training intensity and potential benefits. This challenge highlights the need for adjunctive rehabilitation strategies capable of inducing meaningful muscular and functional adaptations with lower mechanical and metabolic demands (De Labra et al. 2015).

Whole‐body electromyostimulation (WB‐EMS) enables simultaneous activation of large muscle groups through externally applied electrical stimuli and has shown beneficial effects on muscle mass and strength in older and frail populations. In clinical contexts, WB‐EMS is commonly combined with low‐load or assisted movements, making it particularly suitable for individuals with reduced exercise capacity (Kemmler et al. 2021; Yang et al. 2022; de Oliveira et al. 2022; Bloeckl et al. 2022; Kemmler and von Stengel 2013). Nevertheless, evidence regarding its effectiveness in post‐hospitalization transitional care settings remains limited.

Muscle mass has traditionally been assessed using techniques such as DXA, MRI, or bioelectrical impedance, which may be costly, logistically challenging, or influenced by clinical confounders in hospitalized populations (Ling et al. 2011; Tavoian et al. 2019; Bisyri et al. 2022). B‐mode muscle ultrasonography represents a feasible bedside alternative, offering reliable assessment of muscle thickness and sensitivity to short‐term changes (Ticinesi et al. 2017; Paris et al. 2017; López Jiménez et al. 2024; Nies et al. 2022). While previous WB‐EMS trials have primarily focused on appendicular muscle mass assessed by DXA, the effects of WB‐EMS on ultrasound‐derived muscle thickness—particularly of the diaphragm and forearm—remain underexplored.

Including adults aged ≥ 50 years reflects the recognition that frailty is not restricted to advanced age but may emerge earlier in the presence of multimorbidity and prolonged hospitalization. Moreover, the transitional care context provides a clinically relevant window for interventions aimed at preserving muscle structure and facilitating functional recovery before discharge. Therefore, this randomized clinical trial aimed to evaluate the effects of WB‐EMS combined with conventional physiotherapy on rectus femoris muscle thickness, as well as diaphragm and forearm muscle thickness, functional capacity, and frailty in patients admitted to a transitional care unit.

## 2 Methods

### 2.1 Study Design

**FIGURE 1:**
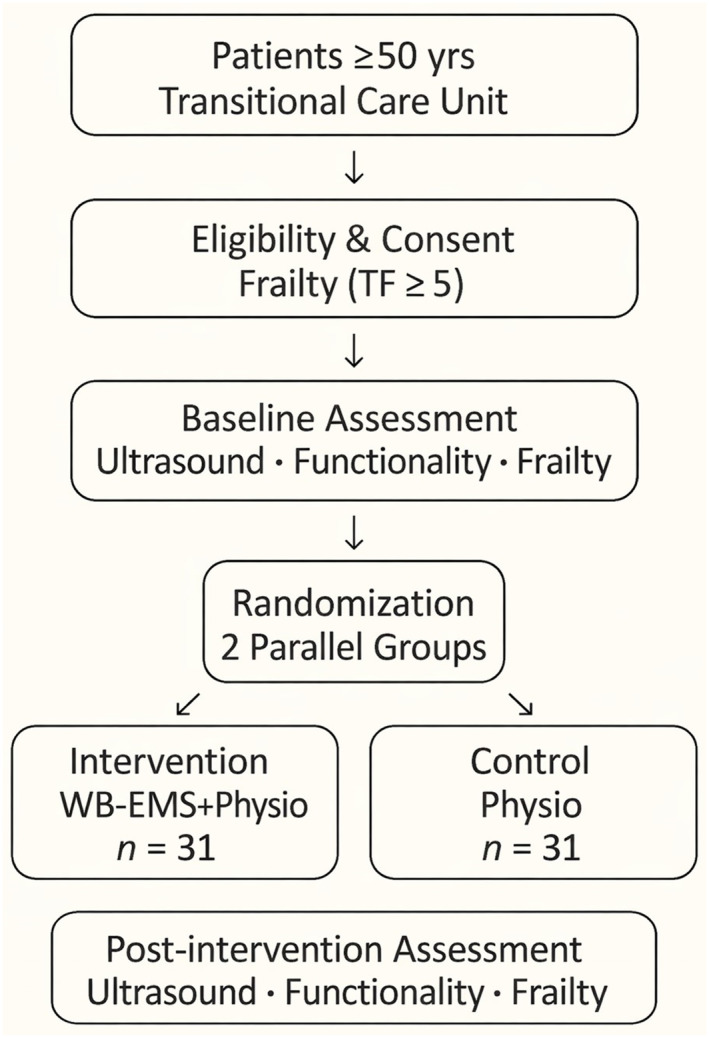
Study design and participant flow.

This study is a two‐arm, parallel, randomized clinical trial with blinded outcome assessment, conducted in accordance with SPIRIT 2025 and CONSORT recommendations (Chan et al. 2025), as illustrated in Figure 1. The trial was prospectively registered in the (ReBEC; RBR‐6DQ3QGN; Brazilian Clinical Trials Registry [ReBEC] 2025) prior to participant enrollment and approved by the Research Ethics Committee of the Federal University of Ceará. Assessments will be conducted at baseline and after 6 weeks of intervention (Table 1).

**TABLE 1:** SPIRIT 2025—Participant timeline (schedule of enrollment, interventions and assessments).

Trial Period	Enrollment	Post‐randomization (intervention phase)	Close‐out/final assessment
Timepoint	T0	T1	T2
		Weeks 1–6 (intervention and monitoring)	Week 6 + 24–72 h
Enrollment:			
Eligibility screen	X		
Informed consent	X		
Demographic and clinical data (pathology, age, sex, weight)	X		
Randomization (blocks)		X (day 0)	
Interventions/comparators:			
WB‐EMS + conventional physiotherapy (intervention group)		X (2 sessions/week × 6 weeks)	
Conventional physiotherapy only (control group)		X (5 sessions/week × 6 weeks)	
Monitoring of fatigue (Borg scale, 3 times per session)		X	
Adverse event monitoring		X	
Assessments:			
Baseline ultrasound (diaphragm, rectus femoris, forearm)	X		
Barthel index (BI)—functionality	X		X (post‐intervention 24–72 h)
Tilburg Frailty Indicator (TFI)—frailty	X		X (post‐intervention 24–72 h)
Ultrasound (muscle thickness re‐evaluation)			X (post‐intervention 24–72 h)
Statistical data recording and verification			X
Data analysis (blinded statistician)			X
Follow‐up/close‐out:			
Reporting of adverse events (if any)			X
Data storage (encrypted USB by external collaborator)			X

*Note:* t_0_ = randomization and baseline assessment; X = activity conducted at the specified timepoint. Intervention phase = 6 weeks (12 WB‐EMS sessions + 30 physiotherapy sessions). Final assessment = 24–72 h after last session.

As this is a single‐center study conducted within a transitional care unit, the findings may be subject to selection bias and limited generalizability; however, this setting represents a clinically relevant context for evaluating rehabilitation strategies during the early post‐hospitalization recovery phase.

## 3 Participants and Eligibility Criteria

### 3.1 Participant Recruitment

Eligible patients will be invited to participate during their stay at the Casa de Cuidados do Ceará, in Fortaleza, Brazil, a transitional care unit that admits patients with diverse clinical etiologies. Eligible participants will be adults aged ≥ 50 years, recently hospitalized and referred to transitional care.

### 3.2 Exclusion Criteria

Participants will be excluded if they have contraindications to electrical stimulation (e.g., implanted pacemakers or defibrillators), uncontrolled cardiovascular conditions, acute infection or fever at enrollment, severe cognitive impairment precluding adherence to study procedures, neuromuscular disorders significantly affecting motor function, open skin lesions at electrode placement sites, or terminal illness. Temporary exclusions (e.g., acute intercurrent illness) will be reassessed for eligibility after clinical stabilization.

### 3.3 Sample Size Calculation

The primary outcome is rectus femoris muscle thickness (mm), assessed by B‐mode ultrasonography at a standardized mid‐thigh location. A conservative standard deviation (*σ*) of 3.0 mm was assumed based on prior studies in older adults (Berger et al. 2015; Meza‐Valderrama et al. 2022).

Based on previous WB‐EMS trials reporting lower‐limb hypertrophy of approximately 8%–20%, a between‐group difference (Δ) of 2.0 mm (≈13% relative increase) was defined as clinically meaningful (Kemmler et al. 2017). Sample size was calculated for a two‐group ANCOVA model adjusting post‐intervention values for baseline, assuming a baseline–post correlation (*ρ*) of 0.60, two‐sided *α* = 0.05, and 80% power. Under these assumptions, 23 participants per group are required. Allowing for 25% attrition, the target sample size was 62 participants (31 per group).

## 4 Procedures

### 4.1 Screening and Initial Assessment

Initially, screening will be conducted among institutionalized individuals in the process of hospital discharge, through the analysis of medical records, to verify whether they meet the inclusion criteria described above. After screening, baseline assessments will include demographic and clinical data, administration of the Barthel Index (BI) and Tilburg Frailty Indicator (TFI), and ultrasonographic evaluation of the diaphragm, rectus femoris, and forearm muscles. The same operator, experienced in neuromuscular disorders and muscle ultrasonography, will perform both the ultrasound assessments and the administration of questionnaires throughout the study.

### 4.2 Randomization and Blinding

Participants will be randomly allocated in a 1:1 ratio to either the WB‐EMS plus physiotherapy group or the physiotherapy‐only group. The randomization sequence will be generated by an independent researcher using a computer‐based random number generator with variable block sizes. Allocation concealment will be ensured through a centralized, web‐based system (REDCap), which releases group assignment only after confirmation of eligibility and completion of baseline assessments.

Outcome assessors and data analysts will remain blinded to group allocation throughout the study. Due to the perceptible nature of the WB‐EMS intervention, participant blinding is not feasible and may introduce performance bias; however, this risk is mitigated by the use of standardized exercise protocols, identical session frequency and duration, equivalent supervision across groups, and blinded outcome assessment.

### 4.3 Unblinding Procedures

Outcome assessors are blinded to group allocation. Unblinding will occur only in case of a serious adverse event where knowledge of the participant's allocation is essential for clinical management. Unblinding will be performed exclusively by the principal investigator through access to secure allocation records. The date, reason, and personnel involved will be documented and reported to the ethics committee if required.

### 4.4 Treatment Protocol

The treatment protocol will be described separately and carried out according to the group to which the study participant belongs, either intervention or control.

### 4.5 Conventional Physiotherapy Protocol

Conventional physiotherapy will be provided to both groups using an identical protocol in terms of exercise selection, session frequency, duration, and supervision. Participants will attend five supervised sessions per week, each lasting 20 min, over a 6‐week period. Table 2 describes the exercises included in the conventional physiotherapy protocol and the number of sets to be performed during the intervention period described above.

**TABLE 2:** Description of the conventional physiotherapy exercise protocol and number of sets.

Exercise protocol description	Number of sets
Shoulder flexion and extension	2
Shoulder adduction and abduction	2
Elbow flexion and extension	2
Hip flexion	2
Hip adduction and abduction	2
Knee flexion and extension	2
Dorsiflexion	2
Plantar flexion	2

The protocol will include low‐load, task‐oriented exercises targeting major muscle groups, mobility, balance, and functional activities, adapted to each participant's functional capacity and clinical status. Exercises may be performed in passive, active‐assisted, or active‐resisted modes as clinically indicated.

Exercise dosage and progression will be matched across groups, and all sessions will be individually supervised by licensed physiotherapists to ensure protocol fidelity, safety, and equivalent training exposure. Perceived exertion will be monitored using the Borg Scale (BS; Borg 1970; Cabral et al. 2017) to guide intensity adjustments within predefined safety limits.

### 4.6 WB‐EMS Protocol

WB‐EMS will be applied exclusively to the intervention group as an adjunct to conventional physiotherapy. Sessions will be performed twice weekly for six consecutive weeks, with each session lasting 20 min.

Electrical stimulation will be delivered in bipolar mode using standardized parameters commonly adopted in clinical WB‐EMS protocols: stimulation frequency of 85 Hz, pulse width of 350 μs, and an intermittent duty cycle of 4 s on and 4 s off. Stimulation will be applied concurrently with low‐load, supervised exercises identical to those performed in the control group.

Stimulation intensity will be individually titrated at the beginning of each session and adjusted throughout the intervention to maintain a perceived exertion between 5 and 6 on the BS, corresponding to moderate exertion. Intensity will be reduced or the session interrupted if predefined safety thresholds are exceeded.

The WB‐EMS protocol is designed to provide consistent neuromuscular stimulation while minimizing mechanical load, ensuring feasibility and safety in frail, recently hospitalized individuals.

Operational details of the WB‐EMS intervention, including device specifications, garment and electrode placement, intensity titration and progression rules, therapist training and competency verification, session fidelity checklists, safety stop‐rules, and permitted or prohibited co‐interventions, are provided in the Supporting Information S1 (S1–S3).

### 4.7 Retention and Follow‐Up

Strategies to maximize retention will include regular contact and coordination with the transitional care team. Reasons for absences, dropouts, and losses to follow‐up will be recorded. Participants who discontinue the intervention will be encouraged to complete follow‐up assessments in accordance with the intention‐to‐treat principle, unless they withdraw their consent. Reasons for discontinuation will be documented (e.g., voluntary withdrawal, medical contraindication, change of residence, loss of contact, or death).

### 4.8 Final Assessment

After the 6‐week intervention period, within 24–72 h, all participants will undergo the final assessment, including all examinations performed at baseline. The final assessment will comprise the BI, TFI and ultrasound assessment of the rectus femoris, diaphragm, and forearm muscles.

### 4.9 Ethical Aspects

This study was approved by the Research Ethics Committee of the University Hospital, Faculty of Medicine, Federal University of Ceará (UFC; protocol no. 57719422.0.3001.5684) and registered in the Brazilian Clinical Trials Registry (ReBEC; RBR‐6DQ3QGN). The study involves minimal risk, including possible joint discomfort, fatigue, or delayed‐onset muscle soreness. Risks will be minimized through professional supervision and appropriate rest intervals. Participants will have access to the research team for clarification or guidance if needed.

### 4.10 Consent Process

Participants will receive verbal and written information about the study and provide written informed consent prior to any procedures. A trained researcher will explain the protocol and answer questions. Participants may withdraw at any time without penalty. Signed consent forms will be securely stored according to institutional guidelines.

### 4.11 Data Management

All study data will be entered directly into REDCap, a secure, encrypted, password‐protected electronic data capture system with audit trails and role‐based access. Personal identifiers will be stored in a separate, restricted‐access field to ensure participant confidentiality. The research team will monitor data accuracy and completeness within REDCap throughout the study.

The statistician, who will remain blinded to group allocation, will have access only to the fully de‐identified final dataset exported from REDCap for analysis. No identifiable information will be included in the analytical files.

### 4.12 Data Monitoring and Safety Procedures

Adverse events (AEs) and serious adverse events (SAEs) will be monitored throughout the study. Interventions will be interrupted according to predefined safety criteria. All AEs and SAEs will be recorded and reviewed weekly by the research team. Serious adverse events will be reported to the ethics committee within 24 h. Detailed definitions and stop rules are provided in the Supporting Information S1 (S2).

## 5 Measures and Instruments

### 5.1 Muscle Ultrasonography

Muscle thickness will be assessed using B‐mode ultrasonography following standardized procedures. Participants will rest in the supine position before image acquisition, and images will be obtained using the transducer positioned perpendicular to the skin surface using minimal pressure. Diaphragm measurements will be obtained at end‐expiration during quiet breathing. Appendicular muscles will be assessed on the dominant limb; in participants with paresis affecting the dominant side, the contralateral limb will be evaluated.

Rectus femoris thickness will be measured at standardized anterior thigh landmarks (López Jiménez et al. 2025; Guerreiro et al. 2017), forearm muscle thickness using standardized landmarks (Abe et al. 2014, 2015), and diaphragm thickness via an intercostal approach, preferentially on the right side (Boussuges et al. 2021). For each site, three images will be obtained and averaged. All assessments will be performed by a trained examiner blinded to group allocation. Measurement reliability will be examined in a subsample; detailed reliability procedures are provided in the Supporting Information S1 (S4).

Rectus femoris thickness was selected as the primary outcome as a sensitive marker of skeletal muscle morphology and sarcopenia‐related changes in older and frail populations. In transitional care and post‐hospitalization settings, ultrasound‐derived structural measures may capture early morphological adaptations preceding detectable changes in functional outcomes, which are often influenced by acute illness, fatigue, and motivational factors.

### 5.2 Function and Frailty Outcomes

Functional capacity and frailty will be assessed using the Barthel Index (BI; Minosso et al. 2010) and the Tilburg Frailty Indicator (TFI; Santiago et al. 2013). Both instruments are widely validated and routinely used in geriatric and rehabilitation research. Changes in BI and TFI scores will complement muscle ultrasonography outcomes by contextualizing physiological adaptations within clinically meaningful domains of daily functioning and multidimensional frailty.

### 5.3 Primary Outcome Analysis

The primary analysis will follow the intention‐to‐treat principle. Post‐intervention rectus femoris muscle thickness will be analyzed using analysis of covariance (ANCOVA), with group as the fixed factor and baseline rectus femoris thickness as a covariate. Age, sex, and baseline frailty (TFI score) will be included as additional covariates. Adjusted between‐group mean differences will be reported with 95% confidence intervals and two‐sided *p* values. Sensitivity analyses will be conducted using linear mixed‐effects models including time (baseline and post‐intervention), group, and their interaction.

### 5.4 Secondary and Exploratory Outcomes

Secondary outcomes include diaphragm thickness, forearm muscle thickness, BI scores, and TFI scores. These outcomes will be analyzed using ANCOVA models analogous to the primary analysis, including the same covariates and adjusting post‐intervention values for baseline measurements. Results for secondary outcomes will be interpreted as supportive and exploratory, without adjustment for multiple comparisons. Exploratory analyses will examine associations between changes in muscle thickness (rectus femoris, diaphragm, and forearm) and changes in functional and frailty measures using Pearson or Spearman correlation coefficients, as appropriate, based on data distribution. These exploratory analyses are hypothesis‐generating and intended to inform future studies rather than provide confirmatory evidence.

### 5.5 Missing Data Handling

Missing outcome data will be handled using Multiple Imputation by Chained Equations under a Missing at Random assumption, in accordance with SPIRIT and CONSORT recommendations. The imputation model will include group allocation, baseline and post‐intervention values of all primary and secondary outcomes, age, sex, and baseline frailty. Twenty imputed datasets will be generated and pooled using Rubin's rules. Primary ANCOVA analysis will be conducted on the multiply imputed datasets to preserve the intention‐to‐treat principle. As sensitivity analyses, linear mixed‐effects models estimated by maximum likelihood will be fitted to all available repeated measures without ad hoc imputation; additional sensitivity analyses will include complete‐case and per‐protocol analyses. The Last Observation Carried Forward will not be used.

## 6 Discussion

Whole‐body electromyostimulation represents a potentially useful adjunct strategy for rehabilitation in individuals with limited tolerance to conventional exercise; however, its physiological and clinical effects in transitional care settings remain incompletely understood. This trial was designed to examine whether WB‐EMS induces measurable changes in muscle thickness during the early post‐hospitalization recovery phase.

Expected physiological adaptations, such as changes in rectus femoris, diaphragm, and forearm muscle thickness, may reflect neuromuscular activation and muscle preservation; nevertheless, the magnitude and consistency of these changes, as well as their translation into functional gains and reduced frailty, remain uncertain. These relationships may be influenced by clinical heterogeneity, baseline frailty severity, comorbidities, and short‐term recovery dynamics typical of transitional care.

From a physiotherapy perspective, ultrasound‐based muscle measures may provide clinically relevant information by capturing early structural adaptations that can precede observable functional improvements. If meaningful associations between muscle thickness changes and functional or frailty outcomes are observed, this may support the selective integration of WB‐EMS as a complementary tool within individualized, multidisciplinary rehabilitation programs for frail, recently hospitalized patients. However, clinical recommendations should be guided by the magnitude of observed effects and their consistency across outcomes.

This study has important limitations. Its single‐center design conducted within a transitional care unit may limit the generalizability of the findings to other clinical contexts and healthcare systems. In addition, the relatively short intervention duration of 6 weeks may not capture longer‐term adaptations in muscle structure, functional recovery, or frailty trajectories. Therefore, inferences regarding sustained or long‐term effects of WB‐EMS should be made with caution.

### 6.1 Protocol Amendments

All protocol amendments will be submitted for review and approval by the institutional Research Ethics Committee prior to implementation. Amendments may include changes to study design, eligibility criteria, outcomes, sample size, procedures, or administrative aspects. Following approval, amendments will be documented in the protocol version history, updated on the trial registry (if applicable), and communicated promptly to all members of the research team. Any substantial changes that may affect participant safety or trial integrity will be reported immediately and managed according to institutional and regulatory requirements.

### 6.2 Ancillary and Post‐Trial Care

Given the minimal risk nature of the intervention, no additional post‐trial care or extended follow‐up is planned. Should any harm or adverse event related to the study occur, participants will receive appropriate clinical evaluation and treatment according to institutional policies at no cost. After the intervention, participants will return to their routine care provided by the health facility.

### 6.3 Implications for Physiotherapy Practice

This study offers evidence‐based insights for physiotherapy researchers investigating novel interventions for frail populations. The WB‐EMS protocol described here may represent a feasible, low‐intensity adjunct to conventional physiotherapy, pending confirmation of clinically meaningful effects to conventional physiotherapy, enhancing muscle structure and functional recovery in hospitalized or recently discharged patients. Findings from this study could help guide the development of standardized and reproducible rehabilitation protocols in geriatric and transitional care settings.

## Author Contributions

André Luis Pinheiro Borges was the principal investigator and lead author, responsible for the conception, design, and overall coordination of the study, as well as the writing and final revision of the manuscript. Jarbas de Sá Roriz Filho served as the academic supervisor (advisor), providing methodological and clinical guidance throughout the study design and manuscript development. Miguel Ângelo Nobre e Souza acted as co‐supervisor (co‐advisor) contributing conceptual insights and critical review of the clinical and physiological aspects of the protocol. Antônio Riquelme Martins Negreiros contributed to data management and elaborated the REDCap database structure for the study. Juliana Nogueira Coelho was responsible for the physiotherapy component of the project, including the execution and adaptation of the intervention protocol, as well as technical contributions to the rehabilitation methodology. The other co‐authors contributed collaboratively to the manuscript preparation, literature review, and conceptual discussions that supported the design and execution of the study.

## Funding

The authors have nothing to report.

## Ethics Statement

This study was approved by the Research Ethics Committee of the University Hospital, Faculty of Medicine, Federal University of Ceará (UFC), under protocol number 57719422.0.3001.5684, in accordance with ethical standards for research involving human participants. It was also registered in the Brazilian Clinical Trials Registry (ReBEC) under protocol number RBR‐6DQ3QGN.

## Consent

Before the inclusion in this study, all participants will sign an informed consent form.

## Conflicts of Interest

The authors declare no conflicts of interest.

## Committees and Oversight

A Data Monitoring Committee will not be formed, as the study involves minimal risk intervention and a small number of participants. Monitoring will be carried out by the researchers themselves.

## Data Sharing Plan

Due to the small sample size and the potential risk of participant re‐identification, the sharing of individual participant data (IPD) is not planned. Aggregated results will be included in the final publication, and the statistical code may be provided upon reasonable request to the Principal Investigator. Data will not be deposited in a public repository.

## Dissemination Plan

The study results will be disseminated through publication in a peer‐reviewed journal and presentations at scientific meetings. A lay summary will be made available to participants upon request. The research team holds full responsibility for data interpretation and dissemination.

## Supporting information


Supporting Information S1

## Data Availability

The data that support the findings of this study are available on request from the corresponding author. The data are not publicly available due to privacy or ethical restrictions.
