# Supplementary material for: Effects of Whole‐Body Electromyostimulation on the Thickness of Appendicular and Respiratory Muscles, Functionality, and Frailty in Patients From a Transitional Care Unit: A Randomized Clinical Trial Protocol
Source: Physiother Res Int. 2026 Mar 16;31(2):e70199. doi: 10.1002/pri.70199 (PMC12989911; doi:10.1002/pri.70199)
Supplement: Supplementary file 1 — Supporting Information S1 [file PRI-31-e70199-s001.docx]

# Supplementary Material – WB-EMS Trial Protocol

## Supplementary Material S1 – WB-EMS Device, Garments, and Electrode Placement

Device model and mode: Miha Bodytec® (Germany), bipolar mode, programmable parameters.

Garment specifications: conductive vest; bilateral arm, thigh, calf straps; pelvic belt; moisture-retaining underlayer; individualized fitting to ensure uniform electrode–skin contact without excessive compression.

Electrode placement map: stimulation of trunk (rectus abdominis, obliques, paraspinals), upper limbs (biceps, triceps, deltoids), and lower limbs (quadriceps, hamstrings, gluteals, calves), following manufacturer anatomical guidelines with symmetrical alignment over muscle bellies.

## Supplementary Material S2 – Intensity Titration, Progression Rules, and Safety Stop-Rules (WB-EMS)

Intensity titration using Borg RPE: start at the lowest perceptible level; progress to Borg 4–5 initially; maintain Borg 5–6 during active exercise; weekly progression of ~5–10% if Borg ≤5 without overexertion.

Upper safety thresholds: reduce intensity if Borg ≥7; stop session if Borg ≥8 or in case of dizziness, nausea, palpitations, chest discomfort.

Physiological stop-rules: sustained tachycardia >140 bpm; frequent ventricular ectopy (>6/min); SBP >180 mmHg or DBP >100 mmHg; chest pain; neurological changes; fever >37.8°C; suspected rhabdomyolysis (severe myalgia, weakness, dark urine).

## Supplementary Material S3 – Therapist Training, Session Fidelity, and Co-interventions

Therapist training and competency: 8-hour training covering WB-EMS operation, electrode placement, safety procedures, AE recognition, standardized Borg application; supervised mock session; competency checklist and brief written assessment prior to authorization.

Session fidelity checklist and documentation: electrode placement; garment hydration; device settings; session duration; completion of physiotherapy exercises; Borg values (start/mid/end); adverse events; protocol modifications; therapist ID; REDCap time-stamped audit trails.

Co-interventions: allowed (routine medical care, conventional physiotherapy for both groups, standard occupational therapy, institutional nutrition); prohibited (additional electrostimulation, high-intensity resistance training, external training programs, hypertrophy supplements such as creatine/protein, manual therapies that may alter muscle thickness); weekly screening for adherence.

## Supplementary Material S4 – Detailed ultrasound reliability procedures

## Equipment and settings Ultrasound assessments will be performed using a Sonosite M-Turbo device equipped with a 6–15 MHz linear transducer. Gain, depth, and focal settings will be standardized across all examinations.

## Pre-imaging standardization Participants will rest in the supine position for 10 minutes before image acquisition. A thick layer of ultrasound gel will be applied, and the transducer will be positioned perpendicular to the skin surface using minimal pressure.

## Image acquisition and processing Three images will be acquired for each anatomical site, and the mean value will be used for analysis.

## Reliability testing Reliability will be examined in a subsample using repeated scans performed 48 hours apart. Intra-rater and inter-rater reliability will be assessed using intraclass correlation coefficients (ICC [2,1]). The standard error of measurement (SEM) will be calculated as SD × √(1 − ICC), and the minimal detectable change at the 95% confidence level (MDC_95) as SEM × 1.96 × √2.

## Site-specific anatomical landmarks Forearm muscle thickness will be measured at 30% of the distance between the ulnar styloid and the olecranon process. Rectus femoris thickness will be measured at two-thirds of the distance between the anterior superior iliac spine and the superior border of the patella. Diaphragm thickness will be obtained via an intercostal window, preferentially on the right side, at end-expiration.
